# Risk perception of genetic effects and mental health among residents of Kawauchi village, 10 years after the Fukushima Daiichi Nuclear Power Plant accident

**DOI:** 10.1093/jrr/rrab108

**Published:** 2022-01-06

**Authors:** Mengjie Liu, Hitomi Matsunaga, Makiko Orita, Yasuyuki Taira, Noboru Takamura

**Affiliations:** Division of Disaster and Radiation Medical Sciences, Nagasaki University Graduate School of Biomedical Sciences, 1-12-4 Sakamoto, Nagasaki, 852-8523, Japan; Department of Global Health, Medicine and Welfare, Atomic Bomb Disease Institute, Nagasaki University, 1-12-4 Sakamoto, Nagasaki, 852-8523, Japan; Department of Global Health, Medicine and Welfare, Atomic Bomb Disease Institute, Nagasaki University, 1-12-4 Sakamoto, Nagasaki, 852-8523, Japan; Department of Global Health, Medicine and Welfare, Atomic Bomb Disease Institute, Nagasaki University, 1-12-4 Sakamoto, Nagasaki, 852-8523, Japan; Department of Global Health, Medicine and Welfare, Atomic Bomb Disease Institute, Nagasaki University, 1-12-4 Sakamoto, Nagasaki, 852-8523, Japan

**Fig. 1:**
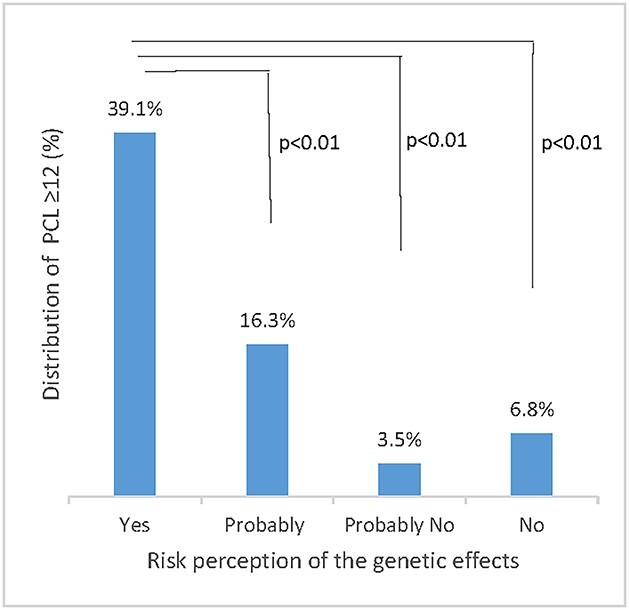
Risk perception of genetic effects in Kawauchi residents with a high PCL-S score (≥12) in 2021.

**Fig. 2:**
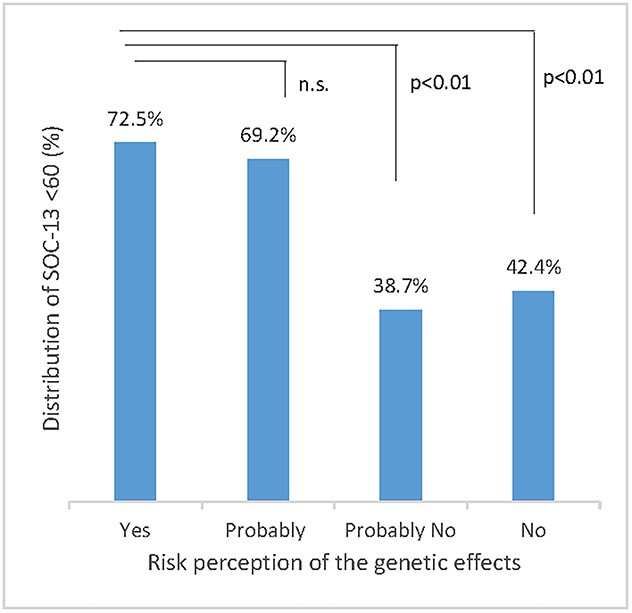
Risk perception of genetic effects in Kawauchi residents with a low SOC-13 score (<60) in 2021.

To the Editor,

Ten years have passed since the accident at the Fukushima Daiichi Nuclear Power Plant (FDNPP) [1]. Kawauchi village is located in Fukushima Prefecture within 30 km of the FDNPP, and most residents were evacuated following the nuclear accident on 15 March 2011. In December 2011, the Prime Minister confirmed a condition equivalent to cold shutdown at FDNPP, and the Mayor of Kawauchi decided that residents of Kawauchi could start to return to some areas in January 2012. After the evacuation order was lifted for all areas in June 2016, residents began returning to their homes, and the rate of residence in Kawauchi increased to about 80% by May 2017 [2]. However, we found that the residents were anxious about the health effects of radiation exposure and the consumption of locally grown food following the nuclear accident. In particular, our studies clarified that the perception of risk of genetic effects was relatively higher among the residents who lived in areas where evacuation orders were lifted [3, 4]. We investigated the risk perception of Kawauchi residents who had residence card at the time of each survey, in 2015 and 2017 regarding genetic effects and again in March 2021 at 10 years after the FDNPP accident. Our definition of the risk perception of genetic effects included genetic changes and heritable diseases. Compared with 49.1% in 2015 and 40.9% in 2017, we found that in 2021, 46.2% of these residents still thought that genetic effects would occur in Kawauchi village due to the FDNPP accident. These results suggest that it is difficult to change people’s risk perception of genetic effects, whether they had returned to their hometown or not. We also investigated the relationship between

risk perception of genetic effects and mental health in Kawauchi residents. We used the indicators of mental health found in the post-traumatic stress disorder checklist-Specific (PCL-S), used in screening for post-traumatic stress disorder (PTSD), to assess the severity of traumatic stress reactions [5, 6]. The sense of coherence-13 (SOC-13) was used to assess capacity to cope with various types of external stress [7, 8]. Among the 374 respondents (203 males and 171 females), 296 (79.2%) were aged ≥60 years. Figs 1 and 2 show that the more that residents thought genetic effects would occur due to the FDNPP accident, the more frequent were symptoms of PTSD and a reduced capacity to cope with stress (analysis by one-way ANOVA followed by Tukey’s multiple comparison test). Furthermore, logistic regression analysis revealed an independent association of a high level of PTSD symptoms (odds ratio, 6.92; 95% confidence interval, 3.26–14.71; *P* value <0.01) and low stress coping level (odds ratio, 3.79; 95% confidence interval, 2.44–5.90; *P* value <0.01) with a higher likelihood of thinking that genetic effects would occur due to the FDNPP accident (Table 1). The UNSCEAR 2020 Report states that there is no credible evidence of genetic effects such as cancer, excess congenital anomalies, stillbirths, preterm deliveries or low birthweight in the general public related to radiation exposure after the FDNPP accident [9]. However, it is clear that even 10 years after the nuclear accident, the perception of risk among residents regarding genetic effects is still polarized [10]. A previous study has suggested that the perception of risk of genetic effects was related to a prolonged decline in mental health [11] as well as a deterioration in the ability to cope with stress. At 10 years after the nuclear accident, Kawauchi village has been almost completely reconstructed. However, these results strongly suggest that it is necessary to continue science-based risk communication with the residents to reduce their anxiety about genetic effects due to the nuclear accident, and thus contribute to the promotion of mental health, particularly among the younger generation who will have children in Fukushima in the near future.

**Table 1:** Logistic regression analysis of the risk perception of genetic effects in Kawauchi residents

Variables	Unit	Model 1		Model 2	
		OR	95%CI	OR	95%CI
Age	≥60/<60	1.48	0.83–2.53	1.98^*^	1.15–3.42
Gender	Female/Male	1.11	0.73–1.71	1.03	0.67–1.59
PCL-S	worse/better	6.92^*^^*^	3.26–14.71	—	
SOC-13	worse/better	—	3.79^*^^*^	2.44–5.90	

Note. OR: Odds Ratio, 95%CI: 95% confidence interval, PCL-S; post-traumatic stress disorder checklist-Specific, SOC-13; the sense of coherence-13.

^*^ *P* < 0.05.

^*^^*^ *P* < 0.01.
